# Shiga Toxin-Producing *Escherichia coli* Isolated from Bovine Mastitic Milk: Serogroups, Virulence Factors, and Antibiotic Resistance Properties

**DOI:** 10.1100/2012/618709

**Published:** 2012-11-19

**Authors:** Hassan Momtaz, Farhad Safarpoor Dehkordi, Taghi Taktaz, Amir Rezvani, Sajad Yarali

**Affiliations:** ^1^Department of Microbiology, College of Veterinary Medicine, Islamic Azad University, Shahrekord Branch, P.O. Box 166, Shahrekord, Iran; ^2^Young Researchers Club, Islamic Azad University, Shahrekord Branch, P.O. Box 166, Shahrekord, Iran; ^3^Department of Clinical Sciences, College of Veterinary Medicine, Islamic Azad University, Shahrekord Branch, P.O. Box 166, Shahrekord, Iran; ^4^College of Veterinary Medicine, Islamic Azad University, Shahrekord Branch, P.O. Box 166, Shahrekord, Iran

## Abstract

The aim of this study was to detect the virulence factors, serogroups, and antibiotic resistance properties of Shiga toxin-producing *Escherichia coli*, by using 268 bovine mastitic milk samples which were diagnosed using California Mastitis Test. After *E. coli *identification, PCR assays were developed for detection of different virulence genes, serogroups, and antibiotic resistance genes of *Escherichia coli*. The antibiotic resistance pattern was studied using disk diffusion method. Out of 268 samples, 73 (27.23%) were positive for *Escherichia coli*, and, out of 73 positive samples, 15 (20.54%) were O26 and 11 (15.06%) were O157 so they were the highest while O111 was not detected in any sample so it was the lowest serogroup. Out of 73 STEC strains, 11 (15.06%) and 36 (49.31%) were EHEC and AEEC, respectively. All of the EHEC strains had *stx1*, *eaeA*, and *ehly*, virulence genes, while in AEEC strains *stx1 *had the highest prevalence (77.77%), followed by *eaeA *(55.55%). Totally, *aadA1 *(65.95%) had the highest while *blaSHV *(6.38%) had the lowest prevalence of antibiotic resistance genes. The disk diffusion method showed that the STEC strains had the highest resistance to penicillin (100%), followed by tetracycline (57.44%), while resistance to cephalothin (6.38%) was the lowest.

## 1. Introduction

Milk is raised as a complete food especially for children and seniors. Its high value for proteins, minerals, fats, and vitamins is undeniable. It is the primary source of nutrition for young mammals before they are able to digest other types of foods. In addition, milk has been processed into various dairy products such as cheese, cream, butter, yogurt, kefir, and ice cream. Daily, millions of people use milk and dairy products. Milk production has a complex process which is done due to activity of bovine mammary glands. The hygienic quality of milking room and animals has a high importance in milk production, but in cases of low hygienic conditions several infections and illnesses occurred in udder tissue. 

Mastitis is considered the most costly disease in dairy herds due to discarded milk and lowered milk production for approximately 80% of costs associated with mastitis, treatment costs, veterinary fees, labor costs early culling, and death [1]. In addition, lowered milk quality due to increased somatic cell count (SCC) in the milk decreases shelf life of milk and cheese making quality [2]. Previous study showed that bacteremia occurs in a significant proportion of cows with severe systemic disease signs [3]. Besides, the quality and hygiene of milk are changed due to mastitis and usually cannot be used for human and animal consumption. Usually in all mastitic cases the amount of milk production reduced. An increase of 25% on world milk demand between 2007 and 2020 is expected [4]. Dairy cattle with acute coliform mastitis, caused primarily by *Escherichia coli *(*E. coli*), exhibit a wide range of systemic disease severity, from mild, with only local inflammatory changes of the mammary gland, to severe, with significant systemic signs including rumen stasis, dehydration, shock, and even death [3].

*E. coli* strains can further be classified according to the presence of virulence factors such as enterohemorrhagic *E. coli* (EHEC), enterotoxigenic *E. coli* (ETEC), enteropathogenic *E. coli* (EPEC), attaching and effacing *E. coli* (AEEC), and Shiga toxin-producing *E. coli* (STEC or VTEC) [5–8]. Several studies showed that STEC strains are an important group for mastitis [9, 10]. 

Previous study showed that, from all serogroups of STEC strains, O55. O111, O124, O119, O114, O26, O157, and O44 are the most prevalent serotypes of *E. coli* isolated from mastitic milk [1]. Numerous studies to identify virulence factors of *E. coli* isolated from cows with clinical mastitis have been conducted [11]. Studies showed that Shiga toxins (*Stx1*, *Stx2*) and *eae* (intimin) are the most important virulence genes in *E. coli* strains isolated from bovine mastitic milk [10, 12, 13]. The cytotoxic necrotizing factor (*CNF*) toxins (*CNF1 *and *CNF2 *genes) are associated with damage to vascular endothelial cells and thrombotic microangiopathy. 

Mainly, treatment of diseases caused by this bacterium often requires antimicrobial therapy; however, antibiotic-resistant strains of bacteria cause more severe diseases for longer periods of time than their antibiotic-susceptible counterparts. Several studies showed that antibiotic resistant in *E. coli* is increasing in these days [14–16]. Therefore, identification of resistance genes of bacteria seems to be so essential in reduction of treatment costs. There is no previous data about detection of virulence genes, serotypes, and antimicrobial resistance of *E. coli *strains isolated from cow in Iran so this present study was carried out for molecular characterization of STEC strains isolated from bovine mastitic milk. 

## 2. Materials and Methods

### 2.1. Sampling and Detecting *E. coli*

Overall 268 bovine mastitic raw milk samples were collected from centers from several geographic regions of Iran, from January 2011 to March 2012. The animals selected for this study were clinically healthy, and the milk samples showed normal physical characteristics. In this study, mastitic milks were identified by the California Mastitis Test (CMT). Samples (5 mL, in sterile glass containers) were transported to the laboratory at ca. 4°C within a maximum of 6–12 h after sampling.

Samples were cultured in MacConkey (MAC) agar (Merck, Germany). Agar plates were incubated at 37°C, and bacterial growth was evaluated after 24 and 48 h. Gram-negative microorganisms were isolated from MAC agar and determined at the species level using cytochrome oxidase, triple sugar iron agar, urea, and indole tests as putatively *E. coli *[17].

### 2.2. DNA Isolation

Bacterial strains were overnight grown in trypticase soy agar (TSA-Merck, German) at 37°C. One colony was suspended in 100 *μ*L of sterile distilled water. After boiling the suspension for 13 min; this was followed by freezing and subsequently centrifuged at 14,000 rpm for 15 min to pellet the cell debris [18]. The supernatant was used as a template for amplification reaction.

### 2.3. Polymerase Chain Reaction

Tables 1, 2, and 3 showed the list of primers which were used for detection of serogroups, virulence genes, and antibiotic resistance genes of STEC strains isolated from mastitic milk samples.  Table 4 showed the PCR conditions for detection of serogroups, virulence genes, and antimicrobial resistance genes in STEC strains isolated from bovine mastitic milk samples. In all PCR reactions, a DNA thermocycler (Eppendorf Mastercycler, Eppendorf-Nethel-Hinz GmbH, Hamburg, Germany) was used. The amplified products were visualized by ethidium bromide staining after gel electrophoresis of 10 *μ*L of the final reaction mixture in 1.5% agarose.

### 2.4. Antimicrobial Susceptibility Testing

Antimicrobial susceptibility tests was performed by the Kirby-Bauer disc diffusion method using Mueller-Hinton agar (HiMedia Laboratories, Mumbai, India, MV1084), according to the Clinical and Laboratory Standards Institute guidelines (CLSI) [19]. After incubating the inoculated plate aerobically at 37°C for 18–24 h in an aerobic atmosphere, the susceptibility of the *E. coli* isolates to each antimicrobial agent was measured and the results were interpreted in accordance with interpretive criteria provided by CLSI (2006). *E. coli* ATCC 25922 was used as quality control organisms in antimicrobial susceptibility determination.

### 2.5. Statistical Analysis

Statistical analysis was performed using *SPSS/16.0* software for significant relationship between incidences of virulence factors and antibiotics resistance genes of *E*. *coli* isolated from various dairy products. Statistical significance was regarded at a *P* value < 0.05.

## 3. Results

In the current study, all *E. coli* colonies were tested by applying PCR method in order to detect 16S rRNA gene of bacterium. According to data, out of 268 bovine mastitic milk samples, 73 (27.23%) were positive for presence of *E. coli* (Table 5). Therefore, it was shown that incidence of *E. coli* in bovine mastitic milk was high. From a total of 73 *E. coli* positive samples, 36 (49.31%) were AEEC and 11 (15.06%) were EHEC subtypes (Table 6). In the other hand, 26 samples (35.61%) were diagnosed as nondetected serotypes (Table 6). Results showed that all of the 11 positive EHEC serogroups had *stx1, eaeA, ehly* virulence genes, while in AEEC serogroups, 28 (77.77%), 5 (13.88%), and 20 (55.55%) samples had *stx1*, *stx2*, and *eaeA* virulence genes, respectively (Table 6). Significant differences (*P* < 0.05) were shown between the presences of AEEC and EHEC serogroups in mastitic milk samples. 

By applying specific primers for detection of STEC serogroups in mastitic milk samples, it was indicated that, out of 73 positive samples for *E. coli*, 15 (20.54%) and 11 (15.06%) samples were positive for incidences of O26 and O157 serogroups while O111, O45, O121, and O128 serogroups had a lower incidences (0.0%, 2.73%, 2.73%, and 2.73%, resp.) (Table 7). In the other hand, 26 (35.61%) samples have been determined as nondetected serogroups. Statistical analysis of data indicated significant differences (*P* < 0.05) between total presence of O26 with O111, O45, O121, and O128 serogroups. 

Distribution of antimicrobial resistance genes in Shiga toxin-producing *Escherichia coli* serogroups isolated from bovine mastitis showed that *aadA1* had the highest prevalence of antibiotic resistance genes (65.95%), followed by *Sul1* (57.44%) and *dfrA1* (55.31%) while *blaSHV* (6.38%) and* CITM *(12.76%) had the lowest incidence of antibiotic resistance genes (Table 8). Besides, O26 serotype had the highest incidence of antibiotic resistance genes while O111 had the lowest incidence of antibiotic resistance genes in *E. coli* isolated from mastitic milk samples. Statistical analysis of data indicated significant differences (*P* < 0.05) between total presence of *aadA1* with *blaSHV*, *CITM* and *cmlA*, *Sul1* with *blaSHV*, *CITM* and *dfrA1 *with *blaSHV* gene.

The disk diffusion method indicated that the STEC serogroups had the highest resistance to penicillin (100%), followed by tetracycline (57.44%), lincomycin (55.31%), streptomycin (48.93%), ampicillin (46.80%), and sulfamethoxazole, (40.42%) but resistance to cephalothin (6.38%), ciprofloxacin (10.63%), and nitrofurantoin (10.63%) was the lowest (Table 9). Significant differences were seen between level of resistance to penicillin with cephalothin, ciprofloxacin, and nitrofurantoin (*P* < 0.05) and tetracycline and lincomycin only with cephalothin.

## 4. Discussion

Our results showed that the STEC strains can cause mastitis in bovine and reduce milk quality for human consumption because some of mastitic cases are subclinical and its diagnosis only is based on the accurate diagnostic tests. Therefore, application of accurate and sensitive assays for detection of subclinical mastitic milks is essential. The rules of milk inspection and control are more important in cases where raw milk is consumed. Several outbreaks of diseases due to *E. coli* [33, 34] showed that inspection and control of food and especially foods with animal origin is a golden key to reducing the risk of contamination. 

There are many studies which showed that the STEC strains are the most prevalent resources for milk-poisoning [7, 35, 36]. Our results showed that the milk of animals with mastitis and especially subclinical mastitis is the main resource for STEC strains. In addition to unsanitary conditions in milk collection and processing, methods of milking, unsanitary conditions of milking machine, and preventing contamination of raw milk with extrinsic factors like staff, insects, and dust, the primary hygiene of milk can be important in presences of STEC strains in milk. Unfortunately, the mechanism of mastitis in bovine herds is not clear. *E. coli* is one of the most frequent bacteria in the environments and, following parturition and the onset of lactation, the immune system is less able to react appropriately to bacterial challenges. Therefore, mastitis occurred due to *E. coli*. A combination of metabolic and hormonal influences may temporarily suppress the immune system in the periparturient period. Additionally, the altered nutritional and energy demands that occur in the periparturient cow during the last trimester and early lactation increase fat metabolism, leading to a buildup of ketone metabolites (ketosis), which also negatively impact the microbicidal properties of circulating neutrophils and increase the cow's susceptibility to mastitis [37]. This temporary and transient immunosuppression increases the cow's susceptibility to opportunistic organisms and increases the likelihood for environmental bacteria to invade the udder and cause mastitis [37, 38]. 

Our results showed that 27.23% of all milk samples were positive for presence of *E. coli* and from these positive samples, O26 serogroup, *stx1* gene, *aadA1* antibiotic resistance gene, and resistance to penicillin antibiotic have the highest frequencies in bovine mastitic milk samples. Previous study [1] showed that, from a total of 181 mastitic milk samples, 57 were positive for *E. coli* and, from these numbers, 19.2%, 15.8%, 12.3%, 12.3%, 10.5%, 7%, 7%, and 3.5% were O55, O111, O124, O119, O114, O26, O157, and O44 serogroups which was inconsistent with our results. Another study [39] showed that, from 40 mastitic milk samples, 77.4% of the isolates belonged to four different O serogroups (O26, O86, O111, and O127) which was in agreement with our results. 

Bean et al. [40] evaluated the “health status” of cows from which isolates were obtained to study virulence genes. In addition to it, in the majority of cases, presence of STEC strains is related to attendance of various virulence genes. Previous study in Egypt [39] revealed that all *E. coli* strains which were isolated from mastitic milk samples had *stx1*, *stx2*, *hylA*, *Flic(h7)*, *stb*, *F41*, *K99*, *sta*, *F17*, *LT-I*, *LT-II*, and *eaeA* virulence genes. Another study confirmed that the *stx2 *and *eaeA* genes were the most prevalent virulence factors in cow's environment that is contaminated by feces, and it is also a frequent cause of bovine mastitis [41]. Study in Turkey indicated that genes encoding Shiga toxins 1 and 2 (*stx1* and *stx2*), intimin (*eaeA*), heat-stable enterotoxin a (*Sta*), and F5 (K99), F41, and F17 fimbriae were the most prevalent virulence factors which were isolated from clinical bovine mastitis cases [9]. 

Previous study from Iran showed that out of 400 samples, 42 specimens were found to be *E. coli* positive and 14 out of 42 isolates carried the *eaeA* gene, 4 isolates were positive for the gene of F41fimbriae and 10 for *stxI* and *stxII* genes [42]. Another investigation on mastitic milk samples during 17 months showed that the most common virulence gene detected was *stx1*, with a prevalence of 31%, followed by *cnf2* (7.5%), *vt2e* (6.25%), and *eaeA* (4%) which was in agreement with our study [40]. 

Some studies indicated that, in addition to virulence genes like *stx1*, *stx2*, *eae*, and *ehly*, the presence of STEC strains is mainly accompanied by attendance of antibiotic resistance genes [11, 43]. Unfortunately studying of the antibiotic resistance genes in *E. coli* strains isolated from mastic milk samples has been done very rare. In one study, of the 123 *E. coli* strains isolated from milk, 15 (10.7%) had a single virulence gene detected by PCR and *CNF2* is the most common virulence gene which was identified [11], but our study showed that the *aadA1* was the most common virulence gene in mastitic milk samples (65.95%). Another study showed that S and P fimbriae, *CNF1*, and *CNF2* are the most common virulence genes in *E. coli* isolated from mastitic milk samples [44]. Despite the presence of these numerous antibiotic resistance genes in *E. coli* strains isolated from mastitic milk samples, developing resistance against common antibiotic drugs is not unexpected. Our results showed that resistance to penicillin, tetracycline, and lincomycin was the highest, while previous study showed that the predominantly observed resistance was to tetracycline (92.2%), streptomycin (90.4%), nalidixic acid (88.3%), amikacin (86.5%), and cephalothin (84.8%). Multidrug resistance was found among 152 isolates (65.8%) [36]. Langoni et al. [45] reported a discrete level of resistance to tetracycline (13.0%) and ampicillin (12.0%) among *E. coli *isolates from bovine mastitis which was lower than our results. Studies performed in the United States indicate that there is no correlation among increased resistance and antimicrobials that are commonly used in dairy cattle for treatment of mastitis [46, 47]. In Switzerland [48], there was no increased antibiotic resistance of mastitis pathogens during the last 20 years, indicating different points of view about this theme. Our results are in contrast with previous study in Switzerland and, in addition to common used antibiotics, the *E. coli* strains which were isolated from mastitic milk samples in our study even had resistance to chloramphenicol and nitrofurantoin. Chloramphenicol and nitrofurantoin are forbidden antibiotics, and the high antibiotic resistance to them in our study indicated the irregular and unauthorized uses of these antibiotics in veterinary treatment in Iran. Unfortunately, veterinarians in many fields of veterinary such as large animal internal medicine, poultry, and even aquaculture use these antibiotics as a basic one. Therefore, in a very short period of time, antibiotic resistance will appear. Therefore, prescription of antibiotics and prescribed antibiotics has the highest effects on providing of antibiotic resistance. In addition to our study, the multiple antibiotic resistance has been reported by Spînu et al. [49], Rangel and Marin [50], Maidhof et al. [51], Mora et al. [52], and Lira et al. [53]. In total the finding which is common between our study and previous researches [54–56] is the high resistance of STEC strains isolated from milk to tetracycline. Therefore, in these situation not only in our country (Iran), nut also all around the world, prescription of tetracycline and penicillin is not effective for the cases of coliforms bovine mastitis. 

On the other hand, in the current situation in Iran, the use of cephalothin, ciprofloxacin, and nitrofurantoin, due to low antibiotic resistance, can be more effective for treatment of diseases caused by *E. coli*. This survey indicated the highest antimicrobial resistance in O26 and O157 serogroups. Totally *E. coli* antibiotic resistance against common antibiotics which are used in veterinary in Iran was so high.

We recommended (i) vaccination of dairy animals (if necessary), observe hygiene in animal's platform, improving methods of milking, checking milking halls in order to detect *E. coli* especially in the animal feces monthly, fumigating milking halls frequently, observing hygiene during milking for prevent *E.coli* mastitis; (ii) using PCR method as an accurate, safe, and fast diagnostic one for accurate detection of pathogens in mastitic milks; (iii) using simple disk diffusion method in order to evaluate the antibiotic resistance of pathogens in mastitis cases; (iv) due to antibiotic resistance especially in *E. coli*, the veterinarians should pay more attention to prescribing the antibiotics; (v) in order to prevent antibiotic resistance in bacteria, we should apply antibiotics more cautiously in animals, detect resistance genes, and finally use different antibiotics periodically. Our results recommended the use of PCR for detection of antibiotic resistance genes of bacteria as a safe, rapid, and accurate method in laboratories. 

## Figures and Tables

**Table 1:** Primers used for detection of virulence genes in Shiga toxin-producing *Escherichia coli *isolated from bovine mastitis*. *

Virulence factor	Primers name	Primer sequences (5′-3′)	Product size (bp)	Reference
Shiga toxin 1 (*stx1*)	Stx1f	AAATCGCCATTCGTTGACTACTTCT	366	[20]
	Stx1r	TGCCATTCTGGCAACTCGCGATGCA		
Shiga toxin 2 (*stx2*)	Stx2f	CGATCGTCACTCACTGGTTTCATCA	282	[20]
	Stx2r	GGATATTCTCCCCACTCTGACACC		
Enteropathogenic attachment and effacement (*eaeA*)	EAE1	TGCGGCACAACAGGCGGCGA	629	[21]
	EAE2	CGGTCGCCGCACCAGGATTC		
Haemolysin (*ehly*)	Hly F	CAATGCAGATGCAGATACCG	432	[22]
	Hly R	CAGAGATGTCGTTGCAGCAG		

**Table 2:** Primers used for detection of Shiga toxin-producing *Escherichia coli* serogroups isolated from bovine mastitis.

Primer name	Sequence	Size of product (bp)	Target gene	Reference
O26-F	CAG AAT GGT TAT GCT ACT GT	423	*wzx *	[23]
O26-R	CTT ACA TTT GTT TTC GGC ATC			
O103-F	TTGGAGCGTTAACTGGACCT	321	*wzx *	[23]
O103-R	GCTCCCGAGCACGTATAAG			
O111-F	TAG AGA AAT TAT CAA GTT AGT TCC	406	*wzx *	[23]
O111-R	ATA GTT ATG AAC ATC TTG TTT AGC			
O145-F	CCATCAACAGATTTAGGAGTG	609	*wzx *	[23]
O145-R	TTTCTACCGCGAATCTATC			
O157-F	CGG ACA TCC ATG TGA TAT GG	259	*wzx *	[23]
O157-R	TTG CCT ATG TAC AGC TAA TCC			
O45-F	CCG GGT TTC GAT TTG TGA AGG TTG	527	*wzx1 *	[24]
O45-R	CAC AAC AGC CAC TAC TAG GCA GAA			
O91-F	GCTGACCTTCATGATCTGTTGA	291	*gnd *	[25]
O91-R	TAATTTAACCCGTAGAATCGCTGC			
O113-F	GGGTTAGATGGAGCGCTATTGAGA	771	*wzx *	[26]
O113-R	AGGTCACCCTCTGAATTATGGCAG			
O121-F	TGGCTAGTGGCATTCTGATG	322	*wzx *	[27]
O121-R	TGATACTTTAGCCGCCCTTG			
O128-F	GCTTTCTGCCGATATTTGGC	289	*galF *	[28]
O128-R	CCGACGGACTGATGCCGGTGATT			

**Table 3:** Primers used for detection of antimicrobial resistant genes in Shiga toxin-producing *Escherichia coli *isolated from bovine mastitis.

Antibiotic	Resistant gene	Sequence	Size of product (bp)	Annealing temperature (°C)	References
Streptomycin	*aadA1 *	(F) TATCCAGCTAAGCGCGAACT	447	58	[29]
		(R) ATTTGCCGACTACCTTGGTC			
Tetracycline	*tetA *	(F) GGTTCACTCGAACGACGTCA	577	57	[29]
		(R) CTGTCCGACAAGTTGCATGA			
Tetracycline	*tetB *	(F) CCTCAGCTTCTCAACGCGTG	634	56	[29]
		(R) GCACCTTGCTGATGACTCTT			
Trimethoprim	*dfrA1 *	(F) GGAGTGCCAAAGGTGAACAGC	367	45	[30]
		(R) GAGGCGAAGTCTTGGGTAAAAAC			
Fluoroquinolone	*qnr *	(F) GGGTATGGATATTATTGATAAAG	670	50	[31]
		(R) CTAATCCGGCAGCACTATTTA			
Gentamicin	*aac(3)-IV *	(F) CTTCAGGATGGCAAGTTGGT	286	55	[32]
		(R) TCATCTCGTTCTCCGCTCAT			
Sulfonamide	*sul1 *	(F) TTCGGCATTCTGAATCTCAC	822	47	[32]
		(R) ATGATCTAACCCTCGGTCTC			
Cephalothin	*blaSHV *	(F) TCGCCTGTGTATTATCTCCC	768	52	[32]
		(R) CGCAGATAAATCACCACAATG			
Ampicillin	*CITM *	(F) TGGCCAGAACTGACAGGCAAA	462	47	[32]
		(R) TTTCTCCTGAACGTGGCTGGC			
Chloramphenicol	*cat1 *	(F) AGTTGCTCAATGTACCTATAACC	547	55	[32]
		(R) TTGTAATTCATTAAGCATTCTGCC			
Chloramphenicol	*cmlA *	(F) CCGCCACGGTGTTGTTGTTATC	698	55	[32]
		(R) CACCTTGCCTGCCCATCATTAG			

**Table 4:** PCR conditions for detection of serogroups, virulence genes and antimicrobial resistance genes in Shiga toxin-producing *Escherichia coli* in bovine mastitis.

Gene	PCR program	PCR volume (50 *μ*L)
O157, O145, O103, O26, O111	1 cycle: 95°C—3 min 30 cycle: 95°C—20 s 58°C—40 s 72°C—30 s 1 cycle: 72°C—8 min	5 *μ*L PCR buffer 10X 1.5 mM MgCl_2_
		200 *μ*M dNTP (Fermentas) 0.5 *μ*M of each primers F and R 1.25 U Taq DNA polymerase (Fermentas) 2.5 *μ*L DNA template

O91, O128, O121, O113, O45	1 cycle: 94°C—6 min 34 cycle: 95°C—50 s 58°C—70 s 72°C—55 s 1 cycle: 72°C—10 min	5 *μ*L PCR buffer 10X 2 mM MgCl_2_
		150 *μ*M dNTP (Fermentas) 0.75 *μ*M of each primers F and R 1.5 U Taq DNA polymerase (Fermentas) 3 *μ*L DNA template

*stx1, stx2, eaeA, ehly *	1 cycle: 95°C—3 min 34 cycle: 94°C—60 s 56°C—45 s 72°C—60 s 1 cycle: 72°C—10 min	5 *μ*L PCR buffer 10X 2 mM MgCl_2_
		200 *μ*M dNTP (Fermentas) 0.5 *μ*M of each primers F and R 1.5 U Taq DNA polymerase (Fermentas) 5 *μ*L DNA template

*aadA1, tetA, tetB, dfrA1, qnr, aac(3)-IV, sul1, blaSHV, CITM, cat1, cmlA *	1 cycle: 94°C—8 min 32 cycle: 95°C—60 s 55°C—70 s 72°C—2 min 1 cycle: 72°C—8 min	5 *μ*L PCR buffer 10X 2.5 mM MgCl_2_
		200 *μ*M dNTP (Fermentas) 0.5 *μ*M of each primers F and R 2 U Taq DNA polymerase (Fermentas) 3 *μ*L DNA template

**Table 5:** Prevalence of *Escherichia coli* isolated from bovine mastitis.

Number of samples	Number of positive samples
268	73 (27.23%)

**Table 6:** Distribution of virulence factors in *Escherichia coli* subtypes isolated from bovine mastitis.

Subtypes	Number of positive samples	Virulence gene
Nondetected	26 (35.61%)	—
EHEC	11 (15.06%)	*stx1, eaeA, ehly: 11 *(100%)
AEEC	36 (49.31%)	*stx1: 28 *(77.77%)
		*stx2: 5 *(13.88%)
		*eaeA: 20 *(55.55%)
		*stx1, eaeA: 23 *(63.88%)
		*stx2, eaeA: 8 *(22.22%)
		*stx1, stx2, eaeA: 5 *(13.88%)

Total	73 (27.23%)	

**Table 7:** Prevalence of Shiga toxin-producing *Escherichia coli* serogroups isolated from bovine mastitis.

Serogroup	O157	O26	O103	O111	O145	O45	O91	O113	O121	O128	Nondetected
Total	11	15	3	—	3	2	6	3	2	2	26
(73)	(15.06%)	(20.54%)	(4.10%)	—	(4.10%)	(2.73%)	(8.21%)	(4.10%)	(2.73%)	(2.73%)	(35.61%)

**Table 8:** Distribution of antimicrobial resistance genes in Shiga toxin-producing *Escherichia coli* serogroups isolated from bovine mastitis.

	*aadA1 *	*tetA *	*tetB *	*dfrA1 *	*qnr *	*aac(3)-IV *	*sul1 *	*blaSHV *	*CITM *	*cat1 *	*cmlA *
O157 (11)	7	6	4	5	6	2	9	1	1	3	2
O26 (15)	12	8	3	7	5	3	6	—	1	5	2
O103 (3)	2	1	2	3	—	2	3	—	—	2	—
O111 (-)	—	—	—	—	—	—	—	—	—	—	—
O145 (3)	2	2	1	2	1	—	1	—	—	1	1
O45 (2)	1	1	1	—	1	—	—	—	1	2	—
O91 (6)	4	3	2	4	2	4	4	2	—	2	—
O113 (3)	2	2	—	3	—	2	2	—	1	1	1
O121 (2)	1	—	1	1	1	—	2	—	2	—	1
O128 (2)	—	—	2	1	1	—	—	—	—	—	—

Total (47)	31 (65.95%)	23 (48.93%)	16 (34.04%)	26 (55.31%)	17 (36.17%)	13 (27.65%)	27 (57.44%)	3 (6.38%)	6 (12.76%)	16 (34.04%)	7 (14.89%)

**Table 9:** Antibiotic resistance properties in STEC serogroups isolated from bovine mastitis (disk diffusion method).

STES Serogroups	P10*	TE30	S10	C30	SXT	GM10	NFX5	L2	CF30	CIP5	TMP5	F/M300	AM10
O157 (11)	11	9	6	4	8	2	4	5	1	2	3	1	6
O26 (15)	15	11	10	6	4	2	3	10	—	1	5	1	8
O103 (3)	3	3	1	1	2	1	—	2	—	—	2	1	2
O111 (-)	—	—	—	—	—	—	—	—	—	—	—	—	—
O145 (3)	3	—	1	1	1	—	—	1	—	—	1	—	—
O45 (2)	2	1	1	2	—	—	1	—	1	—	—	—	1
O91 (6)	6	—	3	1	2	1	2	4	—	1	1	1	3
O113 (3)	3	1	—	1	—	2	—	2	—	—	2	—	1
O121 (2)	2	1	—	1	1	—	—	1	1	—	—	1	1
O128 (2)	2	1	1	1	1	—	1	1	—	1	1	—	—

Total (47)	47 (100)	27 (57.44)	23 (48.93)	18 (38.29)	19 (40.42)	8 (17.02)	11 (23.40)	26 (55.31)	3 (6.38)	5 (10.63)	15 (31.91)	5 (10.63)	22 (46.80)

*In this table, P10: penicillin (10 u/disk); TE30: tetracycline (30 *μ*g/disk); S10: streptomycin (10 *μ*g/disk); C30: chloramphenicol (30 *μ*g/disk); SXT: sulfamethoxazole (25 *μ*g/disk); GM10: gentamycin (10 *μ*g/disk); NFX5: enrofloxacin (5 *μ*g/disk); L2: lincomycin (2 *μ*g/disk); CF30: cephalothin (30 *μ*g/disk); CIP5: ciprofloxacin (5 *μ*g/disk); TMP5: trimethoprim (5 *μ*g/disk); F/M300: nitrofurantoin (300 *μ*g/disk); AM10: ampicillin (10 u/disk).
